# Gender and offender status predicting treatment success in refugees and asylum seekers with PTSD

**DOI:** 10.3402/ejpt.v5.20803

**Published:** 2014-01-30

**Authors:** Håkon Stenmark, Ismail Cuneyt Guzey, Thomas Elbert, Are Holen

**Affiliations:** 1 Department of Neuroscience, Faculty of Medicine, Norwegian University of Science and Technology, Trondheim, Norway; 2 Centre on Violence, Traumatic Stress and Suicide Prevention, Mid-Norway, St. Olav University Hospital, Trondheim, Norway; 3 Department of Research and Development, Division of Psychiatry, St. Olav University Hospital, Trondheim, Norway; 4 Pain Care Unit, St. Olav University Hospital, Trondheim, Norway; 5 Department of Psychology, University of Konstanz, Konstanz, Germany

**Keywords:** Treatment, patient characteristics, gender, offender, trauma, torture

## Abstract

**Background:**

Current knowledge is limited regarding patient characteristics related to treatment outcome of posttraumatic stress disorders (PTSD) in refugees and asylum seekers.

**Objective:**

Gender, torture status, offender status, level of anger, and level of depression were investigated for possible effects on the treatment outcome.

**Method:**

Patient characteristics were explored in 54 refugees and asylum seekers who had completed a treatment program for PTSD. Non-responders (10), those who had the same or higher levels of symptom severity after treatment, were compared with responders, those who had lower symptom severity after treatment (44). Symptom severity was measured by Clinician-Administered PTSD Scale. The non-responders and responders constituted the dichotomous, dependent variable. The independent variables were gender, torture status, offender status, level of anger, and level of depression. T-tests and Exact Unconditional Homogeneity/Independence Tests for 2×2 Tables were used to study the relationship to treatment outcome.

**Results:**

Being male and reporting to have been a violent offender were significantly more frequent characteristics among the non-responders compared to the responders. The levels of pretreatment anger, depression and torture status did not affect the treatment outcome.

**Conclusions:**

The study adds support to findings that females benefit more from treatment of PTSD than males and that violent offenders are difficult to treat within the standard treatment programs.

Refugees and asylum seekers have been identified with high levels of posttraumatic stress disorder (PTSD) (Porter & Haslam, 2005; Sinnerbrink, Silove, Field, Steel, & Manicavasagar, 1997; Turner, Bowie, Dunn, Shapo, & Yule, 2003). As these groups have often experienced extreme traumatization, they often have a high degree of comorbidity; the extensiveness of symptoms may exceed the diagnostic criteria of PTSD. Among clinicians, these patients have been reported to be difficult to treat due to complications such as cultural differences, reliance on interpreters, unemployment, and difficult living conditions (Hjemdal, 2006). Treatment studies underline the complexity of providing help. Some research has even questioned the treatability of severely traumatized refugees, especially the torture survivors (Birck, 2001, 2004; Carlsson, Mortensen, & Kastrup, 2005). Prior studies have shown a large variation in treatment outcome; some patients respond well, while others obtain little or no relief (Halvorsen & Stenmark, 2010; Hinton et al., 2005; Otto et al., 2003; Paunovic & Ost, 2001). In general, the choice of therapeutic techniques and the treatment deliveries have been seen as the salient determinants of the treatment response. In addition, patient characteristics have been considered to be equally relevant (Lambert, 2011).

In this study, we explored some common patient characteristics of refugees and asylum seekers with PTSD in relation to their treatment success. So far, research on patient characteristics has reported conflicting findings; some have found no relevant characteristics (Van Minnen, Arntz, & Keijsers, 2002), whereas others have pointed to sets of patient characteristics that seem likely to influence the outcome. The role of the patients’ gender has been debated. Tarrier, Sommerfield, Pilgrim, and Faragher (2000) found that women with PTSD did better than men, a finding corroborated by Felmingham and Bryant (2012). In other studies, no gender difference was detected (Jaycox, Foa, & Morral, 1998; Marks, Lovell, Noshirvani, Livanou, & Thrasher, 1998). Several studies have reported that the patients’ levels of anger have been negatively correlated with the treatment outcome (Foa, Riggs, Massie, & Yarczower, 1995; Forbes, Creamer, Hawthorne, Allen, & McHugh, 2003; Forbes et al., 2008). Alcohol abuse (Pitman et al., 1991) and a high number of PTSD symptoms prior to treatment (Perconte & Griger, 1991) have also been related to poor outcome. Several authors have emphasized that dissociation may be associated with negative treatment outcomes (Lanius, Brand, Vermetten, Frewen, & Spiegel, 2012; Steuwe, Lanius, & Frewen, 2012; Wolf et al., 2012), although others have not found this to impact the treatment outcome (Hagenaars, Van Minnen, & Hoogduin, 2010; Speckens, Ehlers, Hackmann, & Clark, 2006; Taylor et al., 2003). In the recommendations to clinicians (Zoellner, Feeny, Cochran, & Pruitt, 2003), psychosis, severe dissociation, and offender status have been pointed out to interfere negatively with the treatment of PTSD.

A pertinent assumption has been that asylum seekers whose applications are under evaluation have life situations that are too precarious to be able to benefit from treatment; they should wait until their stay has been granted before treatment may have a fair chance of success (Norredam, Mygind, & Krasnik, 2006). In recent treatment trials, the notion of the untreatability of the asylum seeker with PTSD has not gained support (Neuner et al., 2009; Renner, 2009; Stenmark, Catani, Neuner, Elbert, & Holen, 2013).

Research on patient characteristics related to the treatment outcome of PTSD in refugees and asylum seekers is still in its early stages. To investigate their relevance, this study has drawn data from a larger project including refugees and asylum seekers in a multicenter outcome study on PTSD treatment in Norway. The patient characteristics were examined in relation to treatment outcome by comparing those who got better with those who did not. Based on previous research, characteristics such as gender and level of anger may be expected to correlate with the treatment response. The findings may add to the understanding of the treatment response in refugees and asylum seekers having PTSD, and also help to refine the treatments of these patient groups.

## Method

### Design

The larger study was prospective with three temporal assessment points: pretest, that is, before treatment; posttest at 1 month after treatment; and a follow-up at 6 months after the termination of the treatment. In the current study, the changes in the symptom severity from pretest to 6 months follow-up were used to identify treatment success or failure in relation to the patients’ characteristics. The research trial was approved by the Regional Committee for Medical Research Ethics in Mid-Norway, and by the Norwegian Social Science Data Services.

### Participants

The sample included refugees and asylum seekers who at the time of the current study had completed a randomized controlled multicenter study of PTSD treatment. The participants were both refugees and asylum seekers; they had been referred to treatment in the general psychiatric services of Mid-Norway. Refugees (*N*=63) and asylum seekers (*N*=41) from different nations were screened before admission into the study. The inclusion criteria were age above 18 years, and a PTSD diagnosis according to the DSM-IV criteria. The exclusion criteria were psychotic disorders, current severe substance abuse, or severe suicidal ideations. For a closer description of the comparative treatment study of PTSD, see Stenmark et al. (2013); 81 refugees and asylum seekers were included. Mainly due to obligatory replacements to other parts of Norway, or due to expatriation, 54 finally completed both the treatment and the follow-up at 6 months after treatment. Those patients have been included in the current study. Table 1 shows their sociodemographic and clinical data. There were no significant differences between the intent-to-treat sample (81) and the treatment completers (54) with respect to age, gender, level of education, time spent in exile, asylum status, Hamilton Rating Scale for Depression—HAM-D scores, Clinician-Administered PTSD Scale—CAPS total scores, or their reported number of traumatic events. It is assumed that the nature of the attrition is mostly unaffected by factors that would bias the sample in relation to the outcome.

**Table 1:** Sociodemographic and clinical information of 54 refugees and asylum seekers with PTSD who had completed treatment and 6 months follow-up after treatment

	Participants (*n*=54)	
Demographic information		
Age M (SD)	35.7	(10.2)
Gender M (SD)		
Men	36	(67%)
Women	18	(33%)
Level of completed education, *N* (%)		
No education	5	(9%)
Primary school	19	(35%)
Secondary school	13	(24%)
Vocational school	5	(9%)
University	12	(22%)
Months in exile, M (SD)	60.1	(56.9)
Region of origin, *N* (%)		
Afghanistan	8	(15%)
Iraq	17	(31%)
Middle East (remaining countries)	6	(11%)
Africa	15	(28%)
Other	8	(15%)
Asylum seekers at pretreatment, *N* (%)	19	(35%)
Refugees at pretreatment, *N* (%)	35	(65%)
Change of asylum status during study (%)		
No change	45	83%
Change from asylum seeker to granted asylum	6	11%
Change from asylum seeker to denied asylum	3	6%
Clinical data at pretreatment		
Number of traumatic events (CAPS), M (SD)	8.2	(2.6)
CAPS Score, M (SD)	83.7	(16.1)
HAM-D Score, M (SD)	20.3	(6.2)
Randomized to treatment		
Narrative exposure therapy	33	(61%)
Treatment as usual	21	(39%)

CAPS = Clinician-Administered PTSD Scale; HAM-D = Hamilton Rating Scale for Depression.

### Measures

The PTSD symptom severity was determined by the CAPS (Blake et al., 1995) and computed by the sum of 17 core symptoms. The CAPS is widely recognized as the gold standard for assessment of PTSD. The CAPS has proven to have “excellent psychometric properties” (Blake et al., 1995, p. 81; for a review, see Weathers, Keane, & Davidson, 2001). When CAPS have been translated to languages other than English and used in various ethnic groups, it has likewise demonstrated adequate psychometric properties (Charney & Keane, 2007; Hinton et al., 2005; Paunovic & Ost, 2001). The CAPS has been translated into Norwegian, and back-translated, in cooperation between psychologists specialized in trauma and professional translators. The Norwegian version has, however, not been validated.

MINI International Neuropsychiatric interview was used in the selection of participants to exclude patients with possible comorbid DSM-IV Axis I disorders such as psychosis, substance abuse, and severe suicidal ideations (Sheehan et al., 1998). For the symptom severity of depression, the HAM-D (Hamilton, 1960) was used by computing the sum of the 17 items of the scale. In order to be classified as a torture survivor, the participants had to: (1) report direct experiences of torture on the Life Events Checklist of the CAPS, (2) give an affirmative response to the direct question “Have you ever experienced torture?”, and (3) describe the main methods of torture experienced. The variable related to traumatic events was determined by the number of events endorsed by each patient.

Offender status was detected by item 16 on the Life Events Checklist. To be classified as a violent offender, the patient had to agree to have caused serious injury, harm, or death to someone else. The patients’ levels of anger were calculated from the sum score of section 14, D2 in the CAPS, which specifically addresses anger and irritability. The sociodemographic data included age, gender, country of origin, time in exile, and asylum status.

### Therapists and treatment

Eighteen experienced mental health professionals at eight centers in the public Norwegian general psychiatric services served as therapists. They were clinical psychologists (*N*=5), psychiatrists (*N*=1), psychiatric nurses (*N*=7), occupational therapists (*N*=2), and clinical social workers (*N*=3).

Treatment was carried out in the general psychiatric outpatient clinics and always consisted of 10 sessions of 90-min duration. The treatment was either Narrative Exposure Therapy (NET) (*N*=31) or treatment as usual (TAU) (*N*=24). NET is an exposure-based treatment focusing on chronological working through of the patients’ traumatic events. This is carried out by the patient talking about their life history with a detailed focus on the traumatic events. A written narrative of what the patient has reported is also read aloud at the start of each session, serving as a reiterated exposure to the traumatic events in their right context of time and space. TAU mainly consisted of help with sleep problems, depressive symptoms, problems related to asylum status, and other practical matters. In a previous publication, both treatments proved effective, but patients improved more from NET than from TAU (Stenmark et al., 2013). In this study, patient characteristics were explored independently of the type of treatment.

### Statistics

The treatment completers (*N*=54) were divided into non-responders and responders. The non-responders (*N*=10) encompassed those who showed zero or negative changes, that is, they had the same or even slightly higher symptom scores on the CAPS at 6 months after the termination of treatment. To investigate the patient characteristics related to treatment outcome, the non-responders were compared with all the patients who demonstrated improvements (*N*=44).

T-tests and Exact Unconditional Homogeneity/Independence Tests for 2×2 Tables were used to identify the statistical differences between the non-responders and responders (Berger, 1994, 1996; Berger & Boos, 1994).

The dependent, dichotomous variable was used to show the overall treatment response between the non-responders and responders. The independent variables were gender, torture status, offender status, level of anger, and level of depression. The level of depression and anger were continuous variables, while gender, torture status, and offender status were dichotomous measures.

As the trial only included those patients who had completed pretest, treatment and 6 months follow-up, no patients were missing whole registrations from any of the time-points. One treatment completer missed the item on offender status. This patient (male, treatment responder, torture survivor, received TAU) was therefore not included in the statistical analysis of the offender status. Otherwise, the 54 treatment completers had no missing data on the items used.

*p*-Values under 0.05 were considered significant.

## Results

When comparing the non-responders with the treatment responders, women were more likely to be in the responder group and men more likely in the non-responder group (*p*=0.02). Torture status showed no significant differences between the non-responders and the responders (*p*=0.49). The violent offenders, however, were more likely to be in the non-responder group (*p*=0.02). Pretreatment anger and depression between the non-responders and the responders showed no significant difference.

There was no significant difference between the non-responders and the responders in their reported number of traumatic events, their age, their levels of education, time spent in exile, asylum status, or CAPS total scores as measured before treatment (Table 2).

**Table 2:** Refugees and asylum seekers treated for PTSD (*N*=54): non-responders (*N*=10) compared to all treatment responders (*N*=44)

		Non-responders (*N*=10)	Therapy responders (*N*=44)	
Gender	Women	0	18	(*p*=0.002*)
	Men	10	26	
Torture status	Torture survivor	6	20	(*p*=0.49)
	Not tortured	4	24	
Offender status	Offender	4	2	(*p*=0.009*)
	Non-offenders	6	41	
Depression (HAM-D score)	Mean	20,8	20,3	(*p*=0.49)
	SD	5.1	6.5	
Anger	Mean	4.9	5.1	(*p*=0.47)
	SD	1.1	1.7	

Differences in gender, torture, and offender status were calculated by Exact Unconditional Homogeneity/Independence Tests for 2×2 Tables. Differences in levels of depression and anger were calculated with *t*-tests.

* *P*<0.05

## Discussion

Gender played a major role in relation to treatment success or failure; females improved more from the treatment than males. No association was found between having been tortured and the treatment outcome. However, violent offenders were more frequently represented among the non-responders.

Several factors indicate that the non-responders constitute a specific subgroup; all were male, and many of them reported to have been violent offenders. Since a high proportion of the violent offenders in general are male (Yang & Coid, 2007), it is difficult to determine the relative weight of gender and offender status. A study with a larger number of participants may provide firmer conclusions.

Our findings showing that female refugees and asylum seekers profit more from treatment is, however, in line with other studies showing women to improve more than men after treatment of PTSD. One possible explanation may be that females are better in combining the awareness of affects and cognition. Healthy women and men differ in their encoding and recall of emotional memories (Andreano & Cahill, 2009), and the women may retain emotional memories better over time (Andreano & Cahill, 2009; Bloise & Johnson, 2007; Segal & Cahill, 2009). Given the reported female superiority in emotional recall, women with PTSD may display greater maintenance of responses to exposure-based treatment than men because of their ability to remember and process such memories. Recently, some researchers have shown that estrogen enhances subsequent recall of extinction (Milad et al., 2010). Accordingly, it is possible that women's responses to therapeutic re-exposure of stressors may in part be linked to some aspects of their hormonal activity.

In previous treatment studies, torture survivors have shown conflicting degrees of improvement. In our study, no difference in the treatment outcome was found between those who had experienced torture and those who had not. This suggests higher treatment optimism for the torture survivors.

The current study shows that violent offenders had poorer treatment responses. It must be noted that having been a violent offender in war is rather different from having been a violent offender in a civilian context. Refugees and asylum seekers may have been war veterans, and during the war they may have been forced into situations in which they inflicted serious harm, injury or even death on others. This is rather different from the settings in the civilian life, in which people become violent offenders. The poor treatment response of the offenders in this study may reflect aspects of their personalities and perhaps also complex relationships to re-exposure of traumatic events in therapy. The treatments given were not specifically focused on offender issues. Some authors argue that such problems require specific attention in order to reduce the posttraumatic symptoms. Feeney, Hembree, and Zoellner (2003) call for caution in the delivery of exposure-based PTSD treatments to violent offenders, based on clinical judgment, but limited research evidence. For the violent offenders, they argue, a treatment component involving cognitive restructuring may be required, possibly as an addition to the exposure treatment (Johnston, Ward, & Hudson, 1997; Landenberger & Lipsey, 2005). Kubany (1994) addresses the prominence of guilt in combat-related PTSD. He also argues that specific attention to the combat related guilt is required to reduce the posttraumatic stress symptoms. He suggests cognitive therapy to ameliorate the combat-related guilt, and he has developed a treatment model focusing on changing guilt-related cognitions. In the recent advances of NET, a specialized version has been developed for helping violent offenders with PTSD. Narrative Exposure Treatment for forensic offender rehabilitation (FORNET) has been developed by Elbert et al. Recently, the FORNET has been successfully field tested by Hermenau, Hecker, Schaal, Madl, and Elbert (in press) with ex-combatants from the Democratic Republic of Congo. FORNET not only aims to reduce PTSD symptoms, but also to control aggressive behaviors. Moreover, the role change from a fighter or potential perpetrator to a civilian is specifically addressed and reinforced in this approach.

Especially in the war veterans with PTSD, anger has been found to have a negative influence upon the treatment outcome (Forbes et al., 2003, 2008). Many refugees and asylum seekers have similar backgrounds to those of the war veterans. The expectation of this study was that the pretreatment anger would confound the treatment outcome. The notion that anger would limit treatment success was not supported by this study. As only one item was used to measure anger, some caution is needed in the interpretation of this finding.

The patients’ levels of depression before treatment did not influence the treatment outcome. This is in line with the findings by Hagenaars et al. (2010); they gave prolonged exposure treatment to 60 patients with PTSD, and in particular, they looked at the impact of dissociation and depression upon the outcome. As in our study, the pretreatment levels of depression did not limit the treatment success.

The sample was divided into two groups, using zero as the cut-off point and all therapy responders as the comparison group. With a low N, this procedure provided a simple division that would encompass all of the treatment completers. We acknowledge the difficulties in setting a cut-off value; alternative ways of dividing the sample were considered but discarded when preparing the manuscript. They included a division into three groups with: (1) no change, (2) almost no change, and (3) obvious change. The latter was then estimated by over two standard deviations changes of CAPS score from before the treatment to 6 months after the treatment; this is a recommended cut-off point for clinical changes (Jacobson & Truax, 1991). With a division into three groups, the main findings were the same regarding gender and violent offenders. However, by this procedure the numbers in each group would be very small, and the information about the middle group would be of less informative value.

### Limitations of study

Due to the small sample size, more in-depth statistical analyses were not possible. *p*-Values <0.05 have been chosen as the significance level without adjustment for multi-testing. The sample size made it difficult to use lower *p*-values. In this study only a limited, yet relevant number of characteristics of refugees and asylum seekers were explored. Additional factors may also have influenced the treatment outcome. Furthermore, the study was completed on a sample that received two different treatment approaches. Each approach may have confounded the way the patient characteristics swayed the treatment outcome. The heterogeneity of the refugees and asylum seekers originating from many regions and cultures, have added towards making the findings harder to interpret. The assessment tools were not validated to the languages and cultures of the various asylum seekers or refugees. Due to the multitude of different nationalities, it was not possible to compute a confirmatory factor analysis. The absence of validation to each refugee and asylum seekers’ language and culture is a general limitation in this study, which is shared with several other studies of this population.

Furthermore, the use of interpreters and the differences in the background training of the therapists, may have added to the variability of the outcome. Anger and perpetrator status were measured by single items, which may have reduced the accuracy. Using the general health care system leaves less opportunity to follow strict research regimen in order to control for possible confounders. On the contrary, the use of this general treatment environment may have been a strength for the external validity of the study.

## Conclusions

According to our findings, gender seems to correlate with the treatment outcome of the refugees and the asylum seekers with PTSD. Females seem to profit more from the psychological treatment. Still, it is unclear if the difference is related to gender or to confounding factors. In this study, neither pretreatment anger nor the experience of torture led to any differences in the treatment outcome. Violent offenders mostly belonged to the non-responders group. This finding supports the notion that a special focus on offender issues might be necessary to reduce posttraumatic symptoms in this group. However, further studies with larger samples are required to explore how patient characteristics may relate to therapy outcome in refugees and asylum seekers with PTSD. In addition to the variables explored in this study, the role of dissociation in relation to treatment outcome would be of particular interest.
